# Impact of Tumor Burden on Immune Checkpoint and Conventional Therapy Responses and Outcomes

**DOI:** 10.1158/2767-9764.CRC-25-0327

**Published:** 2025-11-10

**Authors:** Prashanth Gowda, Saiabhiroop R. Govindu, David Hsiehchen

**Affiliations:** 1 Divison of Hematology and Oncology, Department of Internal Medicine, University of Texas Southwestern Medical Center, Dallas, Texas.; 2 Harold C. Simmons Comprehensive Cancer Center, University of Texas Southwestern Medical Center, Dallas, Texas.

## Abstract

Tumor burden may have opposing influences on immunotherapy outcomes by enhancing neoantigen load or imposing barriers to immune responses. Whether tumor burden is a specific determinant of immunotherapy benefit or a general prognostic factor irrespective of drug mechanism remains ambiguous. We performed a *post hoc* individual patient-level data analysis of eight prospective trials, including patients with non–small cell lung cancer, hepatocellular carcinoma (HCC), bladder cancer, and renal cell carcinoma to determine the association between tumor burden and immunotherapy and conventional therapy efficacy. Objective response rates were higher among patients with low tumor burden treated with either atezolizumab or conventional therapies. Low tumor burden was also associated with improved progression-free survival in most cancer types and overall survival in all cancer types irrespective of treatment class. Tumor burden effects were dose-dependent across cancer types. An exception to this was in HCC, in which sorafenib treatment was uniquely associated with improved antitumor effects in high tumor burden cancers. Comparisons of outcomes within tumor burden strata showed that atezolizumab is superior to conventional therapy in improving overall survival but not progression-free survival in both high and low tumor burden patients compared with conventional therapies in non–small cell lung cancer, HCC, and bladder cancer. These findings demonstrate that tumor burden is a histology-agnostic and dose-related prognostic factor rather than an immunotherapy-specific predictive biomarker. Cross-treatment analyses suggest that surrogate endpoints may inadequately account for the detrimental impact of tumor burden, with implications for nonrandomized studies and early-phase trials, particularly in which variations in tumor burden exist in the underlying population.

**Significance:**

Tumor burden is not a specific predictive marker of immunotherapy benefit but has substantial prognostic effects across cancer types and treatment classes. These results highlight the importance and broad applicability of tumor burden as stratification or selection markers in trial design, especially because surrogate endpoints do not wholly capture the detrimental effects of tumor burden on overall survival.

## Introduction

Immune checkpoint inhibitors (ICI) have transformed the therapeutic landscape across many malignancies (1, 2). However, most patients fail to derive long-term benefit, and determinants of ICI outcomes remain incompletely understood. Tumor burden may reduce ICI sensitivity via local and systemic immunosuppression, including stromal immune cell exclusion, cytokine-driven T-cell exhaustion, and metabolic or vascular constraints to immune function (3–7). Early studies in melanoma revealed an association between higher tumor burden and worse ICI outcomes, but evidence in other cancers, such as non–small cell lung cancer (NSCLC), has been conflicted (8–11). Caveats of prior studies include the reliance on small cohorts, retrospective designs, and the lack of comparative analyses of non-ICI therapies, which preclude the separation of predictive and prognostic effects. In addition, emerging evidence indicates that tumor burden is necessary for immunologic priming so that antigenic stimulation is sufficient to initiate a robust and durable immune response (12, 13). Thus, there are contradicting mechanisms underlying the potential interactions between ICI benefit and tumor burden, and whether tumor burden is a specific and widespread predictor of ICI response remains ambiguous.

To robustly assess the impact of baseline tumor burden on ICI response and outcomes, we performed an individual patient–level analysis of eight trials testing atezolizumab in NSCLC, hepatocellular carcinoma (HCC), bladder cancer, and renal cell carcinoma (RCC). This analysis included randomized trials to provide a controlled framework enabling comparisons between atezolizumab and conventional therapies to discern the predictive and prognostic value of tumor burden while minimizing confounding from patient selection and treatment bias.

## Materials and Methods

### Patient population

This analysis was inclusive of all trial patients from the POPLAR (NCT01903993), BIRCH (NCT02031458), OAK (NCT02008227), IMvigor210 (NCT02108652), IMvigor211 (NCT02302807), IMmotion150 (NCT01984242), IMmotion151 (NCT02420821), and IMbrave150 (NCT03434379) trials, encompassing 4,639 patients (2,943 patients treated with atezolizumab and 1,696 patients treated with chemotherapy or targeted therapies) who had complete tumor response and survival data. Individual-level data were accessed through Vivli, a public data-sharing platform. There were no specific criteria used to exclude patients except for subjects for whom clinical data were not accessible due to legal, regulatory, or contractual constraints in data availability. Across all trials, participants could not have received prior ICIs and must have had measurable baseline target lesions.

### Tumor burden assessment and definitions

Tumor burden was determined from the sum of the longest diameters of RECIST target lesions. High tumor burden was defined as a tumor burden greater than the median or the 75th percentile. Low tumor burden was defined as a tumor burden less than the median or the 25th percentile. Atezolizumab was administered at a 1,200 mg fixed dose every 3 weeks on day 1 of each 3-week cycle. Tumor assessments were required to be performed by cross-sectional imaging of the chest, abdomen, and pelvis at baseline and then every 6 or 9 weeks.

### Statistical analysis

Progression-free survival (PFS) and overall survival (OS) were estimated using the Kaplan–Meier method, and survival curves were compared using the log-rank test. Multivariable logistic regression models were used to assess associations between objective response rates and clinical factors, including age, sex, race, and treatment type. Multivariable Cox regression models were used to assess associations between PFS and OS and clinical factors. Analyses were performed using GraphPad Prism version 10 (Dotmatics) and SPSS statistical software version 24 (IBM). An institutionally determined waiver was obtained for the analysis of individual patient–level data because the dataset is deidentified.

## Results

**Figure 1.**
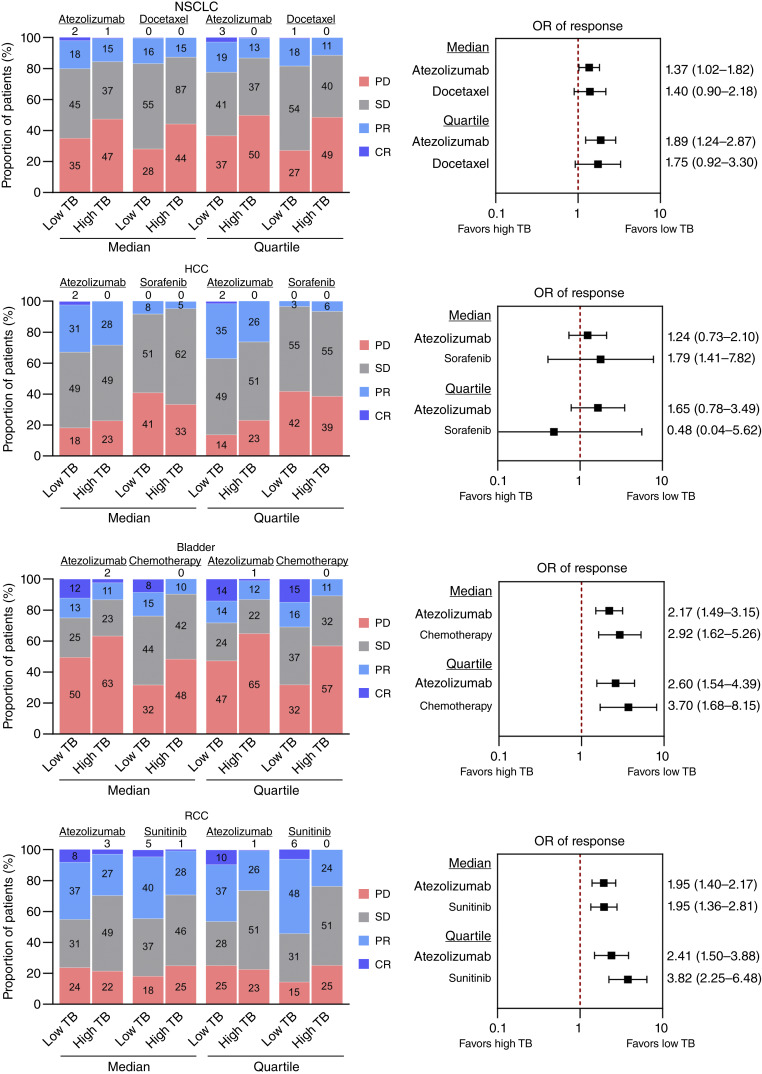
Objective response rates among high and low tumor burden cancers. Distribution of response categories among patients stratified by tumor burden using either median or quartile thresholds for different cancer types and treatments. CR, complete response; PD, progressive disease; PR, partial response; SD, stable disease; TB, tumor burden.

Objective response rates with atezolizumab were greater in low tumor burden patients based on a median or quartile threshold across cancer types (Fig. 1). This was driven by an absence or rarity of complete responses among high tumor burden patients and greater frequencies of partial responses and lower rates of progressive disease in low tumor burden patients (Fig. 1). Similar results were obtained using modified RECIST (Supplementary Fig. S1A and S1B). Among patients treated with conventional therapies, the frequency of objective response rates was greater in high tumor burden patients across cancer types (Fig. 1). Notably, there was an increase in the OR of objective response using quartile versus median thresholds, suggesting dose dependency effects of tumor burden (Fig. 1). Multivariable logistic regression analyses adjusting for demographics and using tumor burden measurements as a continuous variable confirmed a negative association with objective responses (Supplementary Table S1).

**Figure 2.**
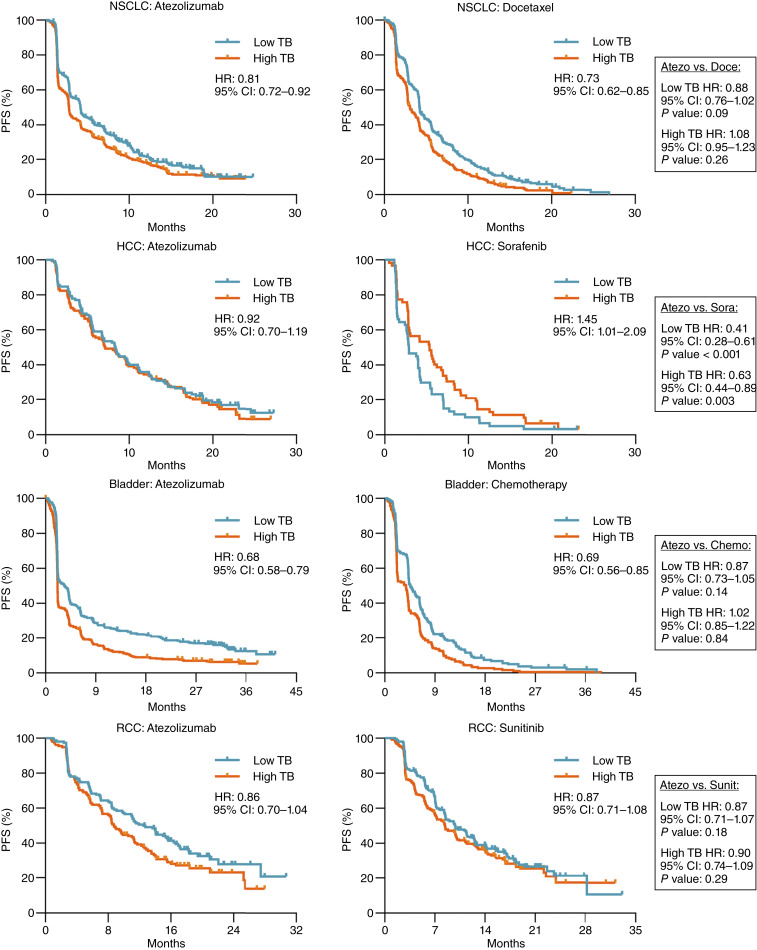
PFS among high and low tumor burden cancers. PFS of patients treated with either atezolizumab or conventional therapies stratified by tumor burden using a median threshold. CI, confidence interval; TB, tumor burden.

Among patients treated with atezolizumab, low tumor burden using a median threshold was associated with worse PFS in NSCLC and bladder cancer (Fig. 2). PFS HRs were less than 1 in HCC and RCC but did not reach statistical significance (Fig. 2). Similar results were observed when PFS was determined according to modified RECIST (Supplementary Fig. S2). Among patients treated with conventional therapies, a low tumor burden was also associated with worse PFS in NSCLC, bladder cancer, and RCC, but an opposite relationship was observed in HCC (Fig. 2). The PFS HR between high and low tumor burden cancers was greater when using quartile thresholds to stratify patients (Supplementary Fig. S3). Multivariable Cox regression analyses adjusting for patient characteristics and using tumor burden measurements as a continuous variable substantiated the association between increasing tumor burden and worse PFS and OS in NSCLC, bladder cancer, and RCC (Supplementary Tables S2 and S3). Comparison of PFS between ICI and conventional therapy within each cancer type, after stratifying individuals by tumor burden, did not show significant differences in outcomes, with the exception of patients with HCC, in which atezolizumab demonstrated improved PFS in both low and high tumor burden patients (Fig. 2; Supplementary Fig. S3).

**Figure 3.**
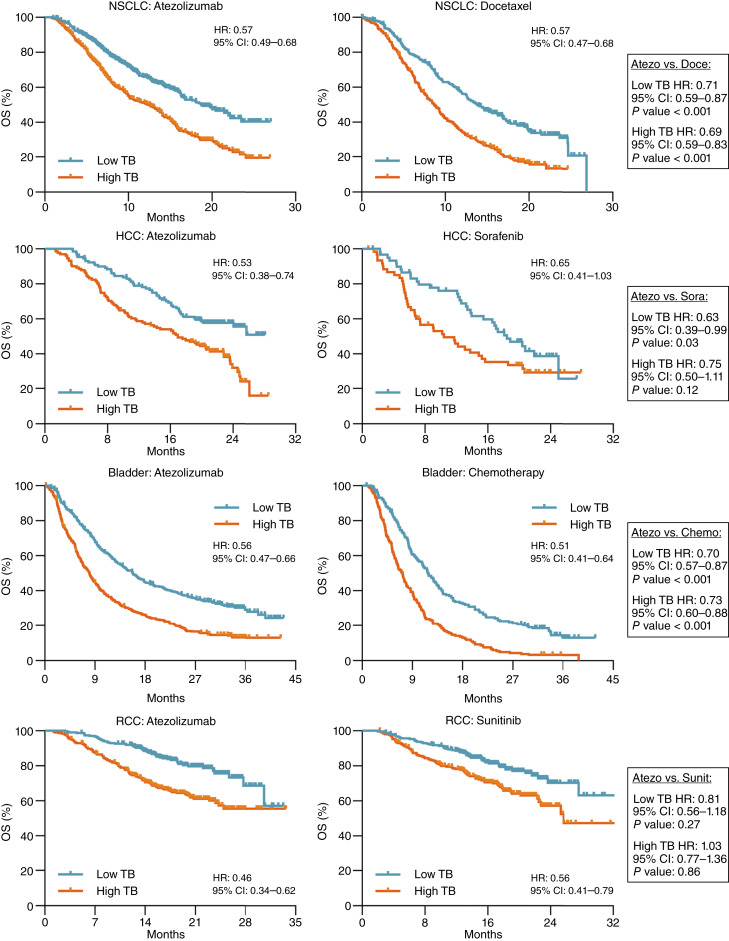
OS among high and low tumor burden cancers. OS of patients treated with either atezolizumab or conventional therapies stratified by tumor burden using a median threshold. CI, confidence interval; TB, tumor burden.

Across all cancer and treatment types, high tumor burden, using a median or quartile threshold, was associated with worse OS (Fig. 3; Supplementary Fig. S4). In fact, OS HRs were nearly identical between atezolizumab and conventional therapies, irrespective of cancer type, and were uniformly decreased when tumor burden was stratified using quartile versus median thresholds (Fig. 3; Supplementary Fig. S4). Multivariable Cox regression analyses confirmed the negative association between tumor burden and OS across cancer types. Comparison of OS between ICI and conventional therapy within individual cancer types after patient stratification by tumor burden showed that OS was improved with atezolizumab compared with conventional therapies in both low and high tumor burden in NSCLC, HCC, and bladder cancer (Fig. 3; Supplementary Fig. S4).

## Discussion

Although high tumor burden patients treated with atezolizumab generally demonstrated lower response rates and worse survival, equivalent associations in strength and direction were observed in patients treated with chemotherapy or targeted therapies. Thus, tumor burden reflects underlying disease biology or host–tumor interactions that negatively affect outcomes regardless of treatment mechanism. Findings from this individual-level data analysis, including randomized trials, highlight the prognostic rather than immunotherapy-specific predictive significance of tumor burden across multiple solid tumors. This contrasts with past studies that included only small patient cohorts or in which matched patients treated with alternative therapies were not examined (3, 9–11, 14). The use of quartile-based stratification magnified the observed associations, suggesting a dose-dependent effect of tumor burden. An exception to this was observed in HCC treated with sorafenib, in which high tumor burden was paradoxically associated with improved PFS but worse OS. This finding could indicate a sorafenib-specific antitumor mechanism in HCC that is more effective against more voluminous disease but is not broadly present enough to overcome the negative prognostic impact of tumor burden.

Comparing outcomes within tumor burden strata showed that atezolizumab treatment was associated with improved OS in NSCLC, HCC, and bladder cancer in both high and low tumor burden patients compared with conventional therapies. This supports the broad efficacy of ICIs in these cancers, regardless of tumor burden, and the importance of tumor burden as a stratification factor in clinical research. Given that differences in PFS between conventional therapy and ICIs within tumor burden strata were not universally observed in cancer types in which similar analyses showed significant OS changes, this cautions that tumor burden is a determinant of long-term outcomes not realized by surrogate endpoints. This suggests that tumor burden may confound the interpretation of early-phase trials—especially those with small samples, nonrandomized designs, or diseases with highly variable tumor burden—and even underlie discrepancies within and across randomized trials when results demonstrate improvements in surrogate endpoints but not OS due to imbalances in tumor burden.

Our results suggest that proposed immune constraints attributed to tumor burden need to be reconsidered (3, 5, 11, 15). A limitation of this study is its reliance on RECIST measurements as a surrogate for tumor burden, which may underestimate tumor burden in some patients. Further research is needed to clarify whether the location of tumor burden, line of therapy, and noncytotoxic and nonimmune drug mechanisms affect the relationship between tumor burden and outcomes. In addition, future investigations are needed to identify the communal mechanisms of tumor burden that allow it to broadly influence outcomes across diverse contexts in a dose-dependent manner and to determine where integrating tumor burden with molecular or immune features may improve patient stratification strategies or personalize treatment selection across drug classes. Given that anti–PD-1/PD-L1 therapies are frequently combined with other drug classes, including other immunotherapies, targeted therapies, and cytotoxic chemotherapies, it also remains to be determined whether multidrug regimens may overcome the prognostic effect of tumor burden.

## Supplementary Material

Supplemental Figure 1 Objective response rates according to immune modified RECIST among high and low tumor burden cancers.

Supplemental Figure 2 Progression-free survival (PFS) according to immune modified RECIST among high and low tumor burden cancers.

Supplemental Figure 3 Progression-free survival (PFS) among high and low tumor burden cancers using quartile thresholds.

Supplemental Figure 4 Overall survival (OS) among high and low tumor burden cancers using quartile thresholds.

Supplemental Table 1 Associations between objective response and clinical factors with tumor burden as a continuous independent variable.

Supplemental Table 2 Associations between PFS and clinical factors with tumor burden as a continuous independent variable.

Supplemental Table 3 Associations between OS and clinical factors with tumor burden as a continuous independent variable.

## Data Availability

This publication is based on research using data from the data contributor Roche that has been made available through Vivli, Inc. Access to individual patient–level data can be requested at https://vivli.org/.
