# Supplementary material for: Impact of Tumor Burden on Immune Checkpoint and Conventional Therapy Responses and Outcomes
Source: Cancer Res Commun. 2025 Nov 10;5(11):1978–83. doi: 10.1158/2767-9764.CRC-25-0327 (PMC12598540; doi:10.1158/2767-9764.CRC-25-0327)
Supplement: Supplemental Figure 1 — Objective response rates according to immune modified RECIST among high and low tumor burden cancers. [file crc-25-0327_supplemental_figure_1_suppsf1.pdf]

Supplemental Figure 1

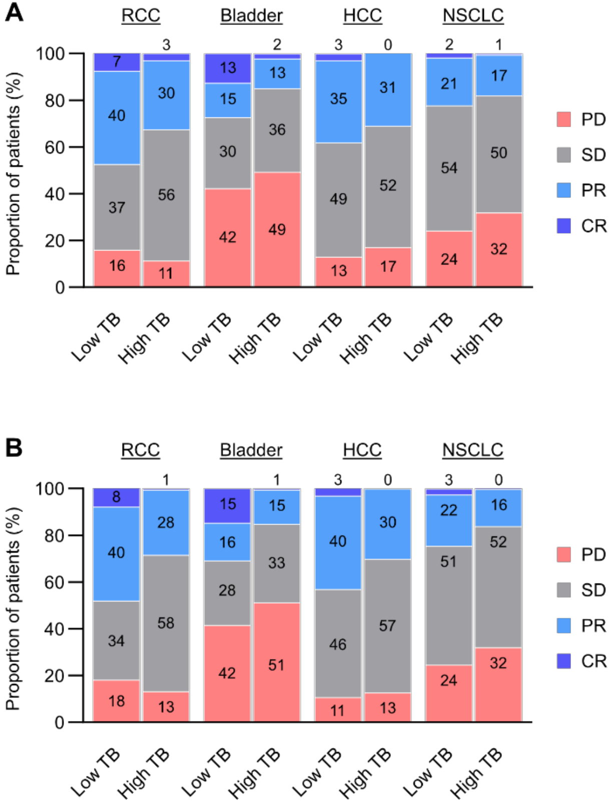

A, Distribution of response categories according to immune modified RECIST among patients treated with atezolizumab stratified by tumor burden using a median threshold for different cancer types. TB, tumor burden.

B, Distribution of response categories according to immune modified RECIST among patients treated with atezolizumab stratified by tumor burden using a quartile threshold for different cancer types. TB, tumor burden.
