# Supplementary material for: Impact of Tumor Burden on Immune Checkpoint and Conventional Therapy Responses and Outcomes
Source: Cancer Res Commun. 2025 Nov 10;5(11):1978–83. doi: 10.1158/2767-9764.CRC-25-0327 (PMC12598540; doi:10.1158/2767-9764.CRC-25-0327)
Supplement: Supplemental Figure 3 — Progression-free survival (PFS) among high and low tumor burden cancers using quartile thresholds. [file crc-25-0327_supplemental_figure_3_suppsf3.pdf]

Supplemental Figure 3

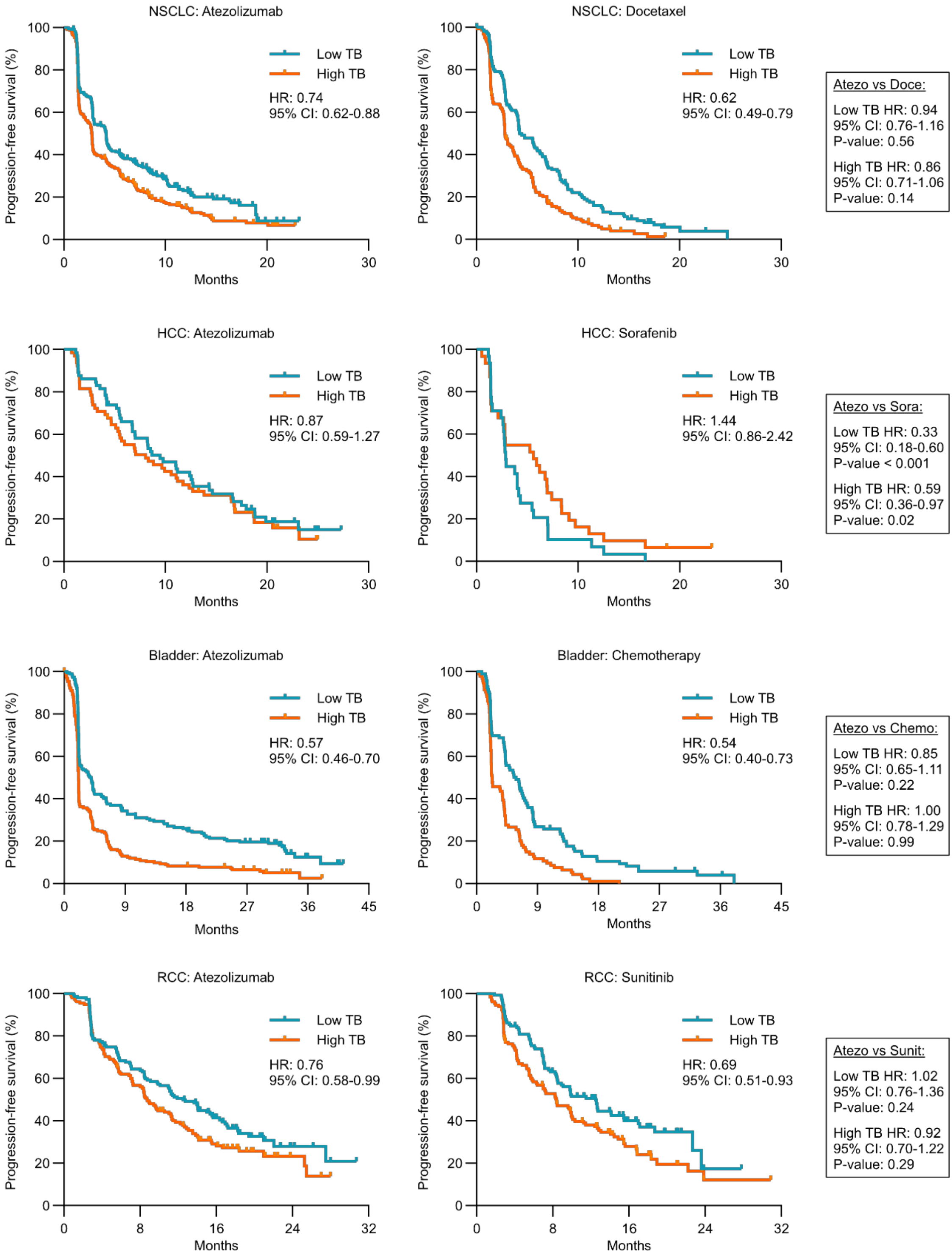

PFS of patients treated with either atezolizumab or conventional therapies stratified by tumor burden using quartile thresholds. TB, tumor burden.
