# Supplementary material for: Impact of Tumor Burden on Immune Checkpoint and Conventional Therapy Responses and Outcomes
Source: Cancer Res Commun. 2025 Nov 10;5(11):1978–83. doi: 10.1158/2767-9764.CRC-25-0327 (PMC12598540; doi:10.1158/2767-9764.CRC-25-0327)
Supplement: Supplemental Table 1 — Associations between objective response and clinical factors with tumor burden as a continuous independent variable. [file crc-25-0327_supplemental_table_1_suppst1.pdf]

Supplemental Table 1. Associations between objective response and clinical factors with tumor burden as a continuous independent variable.

Cancer type	Clinical factors		Adjusted odds ratio	95% CI	P-value
NSCLC	Sum of lesion diameters		0.99	0.99-0.99	0.001
	Treatment	Docetaxel	Reference		
		Atezolizumab	1.24	0.95-1.62	0.11
	Age		1.01	0.99-1.02	0.2
	Sex	Male	Reference		
		Female	1.09	0.86-1.41	0.45
	Race	White	Reference		
		Other	0.95	0.71-1.28	0.75
		Unknown	1.35	0.70-2.61	0.37
HCC	Sum of lesion diameters		0.99	0.99-1.00	0.16
	Treatment	Sorafenib	Reference		
		Atezolizumab	6.41	2.97-13.80	<0.001
	Age		0.99	0.97-1.02	0.6
	Sex	Male	Reference		
		Female	0.57	0.28-1.14	0.11
	Race	White	Reference		
		Other	1.01	0.59-1.70	0.99
		Unknown	0.52	0.17-1.66	0.67
Bladder	Sum of lesion diameters		0.99	0.99-0.99	<0.001
	Treatment	Chemotherapy	Reference		
		Atezolizumab	1.16	0.84-1.62	0.37
	Age		1.01	0.99-1.03	0.08
	Sex	Male	Reference		
		Female	0.57	0.38-0.85	0.01
	Race	White	Reference		
		Other	0.87	0.53-1.42	0.58
		Unknown	1.09	0.68-1.77	0.72
RCC	Sum of lesion diameters		0.99	0.99-0.99	<0.001
	Treatment	Sunitinib	Reference		
		Atezolizumab	1.03	0.80-1.32	0.83
	Age		1	0.99-1.01	0.94
	Sex	Male	Reference		
		Female	0.99	0.76-1.32	0.99
	Race	White	Reference		
		Other	1.21	0.87-1.68	0.26
		Unknown	0.94	0.59-1.49	0.79
