# Supplementary material for: Impact of Tumor Burden on Immune Checkpoint and Conventional Therapy Responses and Outcomes
Source: Cancer Res Commun. 2025 Nov 10;5(11):1978–83. doi: 10.1158/2767-9764.CRC-25-0327 (PMC12598540; doi:10.1158/2767-9764.CRC-25-0327)
Supplement: Supplemental Table 2 — Associations between PFS and clinical factors with tumor burden as a continuous independent variable. [file crc-25-0327_supplemental_table_2_suppst2.pdf]

Supplemental Table 2. Associations between PFS and clinical factors with tumor burden as a continuous independent variable.

Clinical factors				Hazard ratio	95% CI
NSCLC	Sum of lesion diameters		1.003	1.003-1.004	<0.001
	Treatment	Docetaxel	Reference		
		Atezolizumab	0.89	0.81-0.99	0.3
	Age		0.99	0.99-0.99	0.01
	Sex	Male	Reference		
		Female	0.95	0.86-1.04	0.27
	Race	White	Reference		
		Other	1.05	0.94-1.18	0.38
		Unknown	1.02	0.78-1.33	0.89
HCC	Sum of lesion diameters		1	0.999-1.002	0.61
	Treatment	Docetaxel	Reference		
		Atezolizumab	0.52	0.41-0.64	<0.001
	Age		0.99	0.98-1.01	0.33
	Sex	Male	Reference		
		Female	1.16	0.88-1.52	0.29
	Race	White	Reference		
		Other	1.08	0.86-1.36	0.5
		Unknown	1.52	1.03-2.25	0.04
Bladder	Sum of lesion diameters		1.004	1.003-1.005	<0.001
	Treatment	Docetaxel	Reference		
		Atezolizumab	0.97	0.86-1.09	0.59
	Age		0.99	0.98-0.99	0.02
	Sex	Male	Reference		
		Female	1.14	1.00-1.31	0.05
	Race	White	Reference		
		Other	0.98	0.82-1.17	0.81
		Unknown	0.98	0.81-1.18	0.81
RCC	Sum of lesion diameters		1.002	1.001-1.003	<0.001
	Treatment	Docetaxel	Reference		
		Atezolizumab	0.89	0.77-1.02	0.09
	Age		0.99	0.99-1.00	0.06
	Sex	Male	Reference		
		Female	0.94	0.80-1.09	0.42
	Race	White	Reference		
		Other	0.89	0.74-1.08	0.25
		Unknown	1.07	0.82-1.39	0.64
