# Supplementary material for: Impact of Tumor Burden on Immune Checkpoint and Conventional Therapy Responses and Outcomes
Source: Cancer Res Commun. 2025 Nov 10;5(11):1978–83. doi: 10.1158/2767-9764.CRC-25-0327 (PMC12598540; doi:10.1158/2767-9764.CRC-25-0327)
Supplement: Supplemental Table 3 — Associations between OS and clinical factors with tumor burden as a continuous independent variable. [file crc-25-0327_supplemental_table_3_suppst3.pdf]

Supplemental Table 3. Associations between OS and clinical factors with tumor burden as a continuous independent variable.

Clinical factors				Hazard ratio	95% CI
NSCLC	Sum of lesion diameters		1.005	1.005-1.006	<0.001
	Treatment	Docetaxel	Reference		
		Atezolizumab	0.74	0.66-0.83	<0.001
	Age		1	0.99-1.01	0.55
	Sex	Male	Reference		
		Female	0.85	0.75-0.96	0.01
	Race	White	Reference		
		Other	0.83	0.72-0.96	0.01
		Unknown	1.05	0.76-1.46	0.76
HCC	Sum of lesion diameters		1.007	1.005-1.009	<0.001
	Treatment	Docetaxel	Reference		
		Atezolizumab	0.69	0.52-0.89	0.01
	Age		1	0.99-1.02	0.78
	Sex	Male	Reference		
		Female	0.97	0.69-1.37	0.86
	Race	White	Reference		
		Other	0.98	0.74-1.29	0.86
		Unknown	1.1	0.68-1.77	0.69
Bladder	Sum of lesion diameters		1.007	1.005-1.008	<0.001
	Treatment	Docetaxel	Reference		
		Atezolizumab	0.75	0.67-0.85	<0.001
	Age		0.99	0.99-1.00	0.38
	Sex	Male	Reference		
		Female	1.14	0.99-1.32	0.06
	Race	White	Reference		
		Other	0.95	0.79-1.15	0.62
		Unknown	0.98	0.81-1.19	0.83
RCC	Sum of lesion diameters		1.007	1.005-1.008	<0.001
	Treatment	Docetaxel	Reference		
		Atezolizumab	0.89	0.73-1.09	0.27
	Age		1	0.99-1.01	0.43
	Sex	Male	Reference		
		Female	1.05	0.83-1.33	0.68
	Race	White	Reference		
		Other	0.91	0.68-1.22	0.52
		Unknown	1.06	0.70-1.59	0.79
